# Giant Duodenal Perforation Secondary to Malaria in a 5‐Year‐Old Girl: Case Report and Review

**DOI:** 10.1155/cris/5569916

**Published:** 2026-10-21

**Authors:** Ashenafi Shegaw, Mahiderekal Mengistu Berkit, Milki Tufa, Alo Edin

**Affiliations:** ^1^ Department of Surgery Institute of Health Bule Hora University Teaching Hospital Bule Hora University Bule Hora Ethiopia bhu.edu.et; ^2^ School of Medicine Institute of Health Bule Hora University Bule Hora Ethiopia bhu.edu.et; ^3^ Department of Pediatrics and Child Health Institute of Health Bule Hora University Teaching Hospital Bule Hora University Bule Hora Ethiopia bhu.edu.et; ^4^ Department of Epidemiology School of Public Health Institute of Health Bule Hora University Bule Hora Ethiopia bhu.edu.et

**Keywords:** case report, giant duodenal, girl, malaria, perforation

## Abstract

**Background:**

Peptic ulcer disease (PUD) is rare in children, with complications like perforation being especially uncommon. Malaria, a major health burden in endemic regions, is associated with severe complications, but giant duodenal perforation as a direct consequence is remarkably rare. This report presents a 5‐year‐old girl with a giant duodenal perforation secondary to malaria, highlighting the need for prompt diagnosis and intervention.

**Case Presentation:**

A 5‐year‐old girl from a malaria‐endemic area presented with fever, vomiting, and diarrhea. Diagnosed with severe malaria, she developed acute abdominal pain and signs of peritonitis. Imaging confirmed duodenal perforation, which was surgically repaired. Postoperative recovery was successful, and *Helicobacter pylori* infection was ruled out.

**Conclusion:**

Giant duodenal perforation can occur as a rare complication of severe malaria in children without typical peptic ulcer risk factors. Early recognition and timely surgical and antimalarial treatment are critical to improving outcomes in malaria‐endemic settings.

## 1. Introduction

Acute abdomen in pediatric patients poses a significant diagnostic challenge due to the broad spectrum of potential causes and overlapping clinical presentations [1]. While appendicitis remains the most common surgical emergency in children, rarer conditions such as duodenal ulcer perforation can present with similar symptoms, often leading to delayed diagnosis and increased morbidity [2].

Peptic ulcer disease (PUD) is uncommon in the pediatric population, with reported prevalence rates varying by region and demographic factors [2, 3]. Complications like perforation are particularly rare and frequently present diagnostic and therapeutic difficulties [1, 2]. Pediatric PUD is typically associated with risk factors such as *Helicobacter pylori* infection and nonsteroidal anti‐inflammatory drug (NSAID) use; however, these associations are less consistent than in adults, complicating the identification of underlying causes [4, 5].

Malaria continues to be a major health burden in developing countries, disproportionately affecting young children who are vulnerable to severe manifestations predominantly caused by *Plasmodium falciparum* [6]. Although gastrointestinal complications of malaria have been documented, the occurrence of giant duodenal ulcer perforation as a direct complication remains very rare [6]. This case report describes a 5‐year‐old girl presenting with a giant duodenal perforation as an unusual and severe complication of malaria in the absence of typical predisposing factors for PUD. This report aims to emphasize the significance of increased clinical suspicion and prompt management in similar situations.

## 2. Case Presentation

A 5‐year‐old girl from Suro Barguda Woreda, West Guji Zone, Oromia, Ethiopia, residing in a malaria‐endemic area, presented with a 4‐day history of fever accompanied by chills and rigors. The child also had a history of loss of appetite, three to five episodes of vomiting of ingested matter, and the passage of loose stools. The child was evaluated and admitted to Bule Hora Teaching Hospital. Upon initial evaluation, she appeared acutely ill with a blood pressure of 100/60 mmHg, a pulse rate of 12 beats per min (full volume), a respiratory rate of 28 breaths per min, an axillary temperature of 38.6°C, and an oxygen saturation of 92% on room air. HEENT (head, eyes, ears, nose, and throat) examination revealed pale conjunctiva. Physical examination of the other systems was unremarkable.

Laboratory investigations showed a complete blood count with a white cell count of 18,000 cells/µL, a hemoglobin level of 8.7 g/dL, and a platelet count of 42,000 cells/µL. Blood film results showed the trophozoite stage of *P. falciparum*. Her random blood sugar level was 156 mg/dL, and her blood group was B‐positive. With a diagnosis of severe malaria, the patient was started on artesunate 2.4 mg/kg at 0, 12, and 24 h, along with maintenance fluids. The patient was closely monitored in the emergency department. After 24 h of treatment, the surgical unit was consulted after the patient developed abdominal pain, distention, and bilious vomiting.

On re‐evaluation, the patient appeared lethargic and dehydrated. The vital signs were as follows: blood pressure was 90/60 mmHg, pulse rate was 127 beats/min; respiratory rate was 42 breaths/min; temperature was 38.5°C; and oxygen saturation was 89% on room air. Examination of the HEENT revealed pale conjunctiva, dry lips, and buccal mucosa. Abdominal examination revealed a distended abdomen with guarding and rigidity, and direct and rebound tenderness were present in all quadrants.

Abdominal ultrasound was performed and demonstrated free peritoneal fluid with air foci, suggestive of viscus perforation. Plain abdominal radiography revealed the presence of free gas under both domes of the diaphragm (Figure 1).

**Figure 1:**
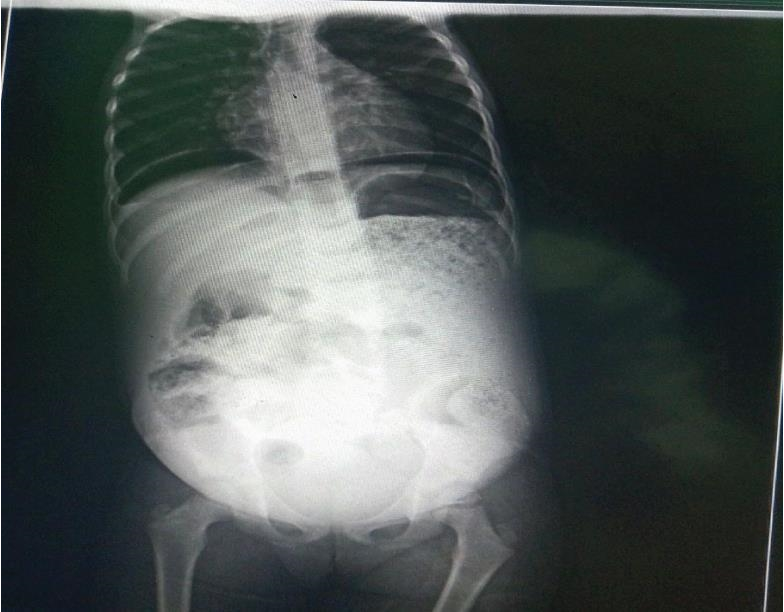
Plain abdominal radiography showing the presence of free gas under both domes of the diaphragm.

With a clinical impression of generalized peritonitis secondary to a perforated viscus, the patient was resuscitated and started on intravenous ceftriaxone and metronidazole therapy. One unit of blood was prepared and administered. The patient was immediately taken to the operating theater for an emergency exploratory laparotomy.

During the operation, a gush of air was released upon opening the peritoneum, and ~1000 mL of gastrointestinal contents were sucked out from the peritoneal cavity. A 2 cm × 2 cm perforation was identified in the anterior first part of the duodenum (Figure 2). The perforation was repaired using an omental pedicle patch, and the abdomen was copiously lavaged with warm saline. A peritoneal drain was placed in the subhepatic space, and the abdomen was closed in a layered fashion.

**Figure 2:**
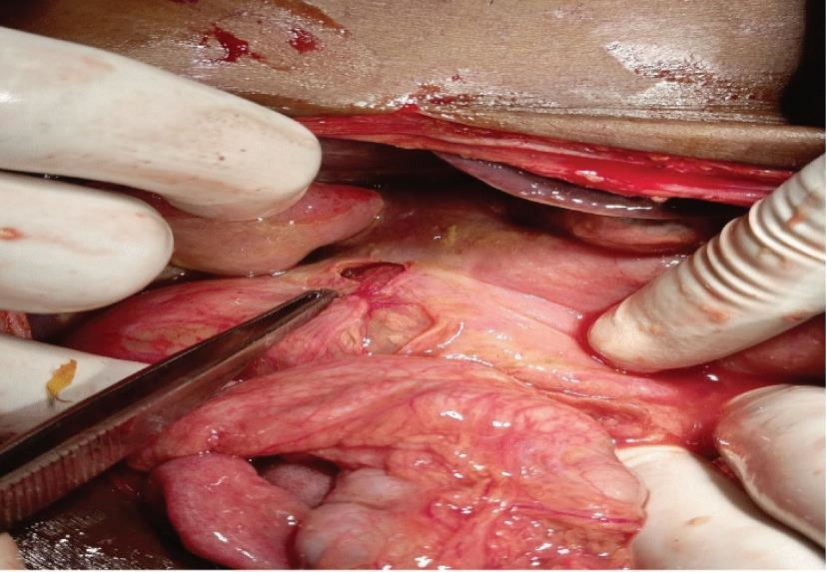
Intraoperative image showing a 2 cm × 2 cm perforation that was identified in the anterior first part of the duodenum.

Postoperatively, the child awakened smoothly from anesthesia and was transferred to the postanesthetic care unit. However, from the immediate postoperative period until the second postoperative day, the child experienced oxygen desaturation and was maintained on intranasal oxygen therapy. Subsequently, the patient’s condition improved, the nasogastric tube was removed on the second postoperative day, and oral sips were initiated. The drainage tube was removed on the fifth postoperative day, and antimalarial medication was completed. The patient was discharged after receiving 10 days of intravenous ceftriaxone and metronidazole. *H. pylori* stool antigen testing, performed on the day of discharge, was negative.

## 3. Discussion

The occurrence of giant duodenal perforation in this 5‐year‐old patient with severe malaria highlights a rare but critical complication of malaria in pediatric populations. While PUD is predominantly linked to *H. pylori* infection and certain demographic factors such as blood group O and male gender, this case deviates from these common associations, suggesting alternative pathogenic mechanisms [7]. The absence of *H. pylori* infection and the patient’s blood group B status, along with her young age and female gender, indicate that the ulceration and subsequent perforation likely resulted from secondary association rather than primary PUD [7, 8].

Stress‐related mucosal disease arises in critically ill patients through multiple overlapping mechanisms, including splanchnic vasoconstriction, reduced mucosal blood flow, acid‐induced damage, and oxidative stress [9]. Experimental studies have also identified reactive oxygen species and activation of nuclear factor‐κB as key contributors to stress‐induced gastric injury [10].

Secondary PUD in children is often precipitated by systemic stressors, including severe infections, shock, or inflammatory states, which can disrupt mucosal integrity and impair healing [11–14]. The pathogenesis of duodenal perforation in malaria is multifactorial, likely involving a combination of direct parasitic injury to the duodenal mucosa, shock and dehydration due to gastrointestinal fluid loss, and physiological stress induced by severe illness [5, 11, 13]. This pathophysiological cascade may increase the risk of ulcer development and, in rare cases, perforation. Evidence about duodenal perforation associated with malaria is scarce. Our case report, however, is in line with a study from Metema, Gondar, Ethiopia, that found a 4‐year‐old child had a giant duodenal perforation linked to a malaria complication [15], while a study from Nepal reported a 5‐year‐old boy who had a duodenal perforation as a result of severe malaria [16]. A similar instance of duodenal perforation in a 4‐year‐old boy was reported from India [17]. Ghana’s Kintampo Municipal Hospital reported a case involving a 1‐year‐old infant, highlighting the possibility of this problem even in younger patients [8].

This case report presents a rare and significant occurrence of giant duodenal perforation secondary to severe malaria in a pediatric patient, highlighting an uncommon but critical gastrointestinal complication associated with malaria. While malaria predominantly manifests with febrile illness and hematologic complications, its association with PUD and subsequent perforation remains scarcely reported, especially in children [5]. The absence of typical risk factors for PUD in this patient, such as *H. pylori* infection, blood group O, medication use, or underlying comorbidities, strengthens the likelihood that the duodenal perforation was likely due to the complication of severe malaria.

The clinical presentation of peritonitis with abdominal distension, guarding, and rigidity in the context of severe malaria should prompt clinicians to consider gastrointestinal perforation as a differential diagnosis, particularly in endemic regions where malaria is prevalent.

Surgical intervention remains the cornerstone of management for duodenal perforation. In this case, exploratory laparotomy with primary repair using an omental pedicle patch and thorough peritoneal lavage was successfully performed, followed by broad‐spectrum antibiotics plus targeted antimalarial therapy. Early surgical management combined with appropriate treatment of the underlying malaria infection is crucial to reduce morbidity and mortality associated with this complication.

This report underscores the need for heightened clinical vigilance for abdominal emergencies in children with severe malaria as delays in diagnosis and treatment of perforation can lead to poor outcomes. It also broadens the clinical spectrum of malaria‐related complications, advocating for the inclusion of secondary PUD and gastrointestinal perforation in the differential diagnosis of acute abdomen in pediatric patients from malaria‐endemic areas.

Future studies are reasonable to better understand the incidence, pathogenesis, and optimal management strategies for malaria‐associated gastrointestinal complications. Meanwhile, awareness among healthcare providers can facilitate timely diagnosis and intervention, improving the prognosis in affected children.

## 4. Strengths

The report details a particularly uncommon case of giant duodenal perforation resulting from severe malaria in a young child, a complication rarely observed in pediatric cases. This underscores a distinct clinical situation that enhances the comprehension of gastrointestinal complications linked to malaria. The case report provides new evidence by recording a giant duodenal perforation in a child with severe malaria, in the absence of the usual risk factors for PUD. It broadens the clinical range of malaria complications, showcasing a rare yet serious gastrointestinal emergency that necessitates immediate surgical and medical treatment. By comparing this case with a few similar reports from various endemic areas, it emphasizes the importance of acknowledging malaria as a possible contributing factor to duodenal perforation in children.

## 5. Limitations

Being a single case report, the findings have limited generalizability and cannot establish the causality between malaria and giant duodenal perforation.

The lack of detailed histopathological examination of the duodenal tissue limits understanding of the exact pathological mechanisms involved.

The absence of long‐term follow‐up data restricts assessment of the patient’s recovery trajectory and potential late complications.

## 6. Conclusions

This case report highlights a rare but serious complication of severe malaria in a pediatric patient, indicating that giant duodenal perforation can occur even in the absence of common risk factors for PUD. Prompt recognition of acute abdominal signs in children with severe malaria is essential to facilitate timely surgical intervention and appropriate antimalarial treatment, thereby reducing morbidity and mortality. Increased clinical awareness of gastrointestinal complications in malaria‐endemic regions is crucial for improving outcomes in affected children.

**Nomenclature**
PUD: Peptic ulcer disease
NSAID: Nonsteroidal anti‐inflammatory drug
cm: Centimeter
Hgb: Hemoglobin.

## Author Contributions

Ashenafi Shegaw contributed to conceptualization, study design, data acquisition, drafting the article, data analysis and interpretation, and critical revision for significant intellectual content. Mahiderekal Mengistu Berkit contributed to data analysis and interpretation, drafting the article, and critical revision for significant intellectual content. Milki Tufa contributed to data acquisition, analysis, and critical revision for significant intellectual content. Alo Edin contributed to conceptualization, analysis, drafting the article, data analysis and interpretation, and critical revision for significant intellectual content.

## Funding

No grant has been received from any funding agency.

## Disclosure

All authors have approved the final version for submission.

## Ethics Statement

The Institutional Review Board (IRB) of Bule Hora University waived the requirement for ethical approval for this study. Written informed consent was obtained and signed by the patients’ parents for publication and the use of any accompanying images. A copy of the written consent is available from the corresponding author upon request.

## Consent

Written informed consent was obtained and signed by the patients’ parents for publication and the use of any accompanying images. A copy of the written consent is available from the corresponding author upon request.

## Conflicts of Interest

The authors declare no conflicts of interest.

## Supporting Information

Additional supporting information can be found online in the Supporting Information section.

## Supporting information


**Supporting Information** File S1: CARE checklist for giant duodenal perforation secondary to malaria in a 5‐year‐old girl: case report and review.

## Data Availability

All necessary information is included within the article.
