# Supplementary material for: Giant Duodenal Perforation Secondary to Malaria in a 5‐Year‐Old Girl: Case Report and Review
Source: Case Rep Surg. 2026 Oct 21;2026:5569916. doi: 10.1155/cris/5569916 (PMC13685499; doi:10.1155/cris/5569916)
Supplement: Supplementary file 1 [file CRIS-2026-5569916-s001.pdf]

CARE Checklist of information to include when writing a case report

CARE case report guidelines

(cc) BY-NC-ND

Topic	Item	Checklist item description	Reported on Line

Title 1 The diagnosis or intervention of primary focus followed by the words "case report" "Giant Duodenal Perforation Secondary to Malaria in a 5-Year-Old Girl: Case Report and Review" **Line: 1**

Key Words 2 2 to 5 key words that identify diagnoses or interventions in this case report, including "case report" Giant Duodenal, Perforation, Malaria, Girl, Case Report. **Line: 33**

Abstract (no references)

3a Introduction: What is unique about this case and what does it add to the scientific literature? Peptic ulcer disease is rare in children, with complications like perforation being especially uncommon. Malaria, a major health burden in endemic regions, can cause severe complications, but giant duodenal perforation as a direct consequence is exceedingly rare. This report presents a 5-year-old girl with a giant duodenal perforation secondary to malaria, highlighting the need for prompt diagnosis and intervention. **Line: 21-25**

3b Main symptoms and/or important clinical findings . A 5-year-old girl from a malaria-endemic area presented with fever, vomiting, and diarrhea. **Line: 26-27**

3c The main diagnoses, therapeutic interventions, and outcomes Diagnosed with severe malaria, she developed acute abdominal pain and signs of peritonitis. Imaging confirmed duodenal perforation, which was surgically repaired**. Line : 27-28.**

3d Conclusion—What is the main "take-away" lesson(s) from this case? Giant duodenal perforation can occur as a rare complication of severe malaria in children without typical peptic ulcer risk factors. Early recognition and timely surgical and antimalarial treatment are critical to improving outcomes in malaria-endemic settings. **Line: 30-32**

Introduction 4 One or two paragraphs summarizing why this case is unique (**may include** references) Malaria continues to be a major health burden in developing countries, disproportionately affecting young children who are vulnerable to severe manifestations predominantly caused by Plasmodium falciparum(Chaudhary, 2013). Although gastrointestinal complications of malaria have been documented, the occurrence of a giant duodenal ulcer perforation as a direct complication remains exceedingly rare(Chaudhary, 2013). This case report describes a 5-year-old girl presenting with a giant duodenal perforation as an unusual and severe complication of malaria, in the absence of typical predisposing factors for PUD. This report aims to emphasize the significance of increased clinical suspicion and prompt management in similar situations. **Line: 48-55**

Patient Information

5a De-identified patient specific information. **Not applicable**

5b Primary concerns and symptoms of the patient. A 5-year-old girl from Suro Barguda Woreda, West Guji Zone, Oromia, Ethiopia, residing in a malaria-endemic area, presented with a four-day history of fever accompanied by chills and rigors. The child also had a history of loss of appetite, three to five episodes of vomiting of ingested matter, and passage of loose stools. **Line: 57-60.**

5c Medical, family, and psycho-social history including relevant genetic information. **Not applicable**

5d Relevant past interventions with outcomes. **Not applicable**

Follow-up and Outcomes

Clinical Findings

Timeline

Diagnostic Assessment

Therapeutic Intervention

suggestive of viscus perforation. Plain abdominal radiography revealed the presence of free gas under both domes of the diaphragm. Line: 79-81

6 Describe significant physical examination (PE) and important clinical findings

8b Diagnostic challenges (such as access to testing, financial, or cultural). **Not applicable**

8c Diagnosis (including other diagnoses considered) On re-evaluation, the patient appeared lethargic and dehydrated. The vital signs were as follows: blood pressure was 90/60 mmHg, pulse rate, 127 beats/min; respiratory rate, 42 breaths/min; temperature, 38.5°C; and oxygen saturation, 89% on room air. Examination of the head, eyes, ears, nose, and throat revealed pale conjunctiva, dry lips, and buccal mucosa. Abdominal examination revealed a distended abdomen with guarding and rigidity, and direct and rebound tenderness was present in all quadrants.

7 Historical and current information from this episode of care organized as a timeline

Abdominal ultrasound was performed and demonstrated free peritoneal fluid with air foci, suggestive of viscus perforation. Plain abdominal radiography revealed the presence of free gas under both domes of the diaphragm. With a clinical impression of generalized peritonitis secondary to perforated viscus, the patient was resuscitated and started on intravenous ceftriaxone and metronidazole therapy. One unit of blood was prepared and administered. The patient was immediately taken to the operating theater for an emergency exploratory laparotomy. **Line:73-88.**

8d Prognosis (such as staging in oncology) where applicable. **Not applicable**

9a Types of therapeutic intervention (such as pharmacologic, surgical, preventive, self-care) . The patient was immediately taken to the operating theater for an emergency exploratory laparotomy. During the operation a gush of air was released upon opening the peritoneum, and approximately 1000ml of gastrointestinal contents were sucked out from the peritoneal cavity. A 2 × 2 cm perforation was identified in the anterior first part of the duodenum (Fig. 2). The perforation was repaired using an omental pedicle patch, and the abdomen was copiously lavaged with warm saline. A peritoneal drain was placed in the subhepatic space, and the abdomen was closed in a layered fashion. **Line: 87-94**

8a Diagnostic testing (such as PE, laboratory testing, imaging, surveys). Abdominal ultrasound was performed and demonstrated free peritoneal fluid with air foci,

9b Administration of therapeutic intervention (such as dosage, strength, duration). The patient was discharged after receiving 10 days of intravenous ceftriaxone and metronidazole**. Line: 103-104.**

9c Changes in therapeutic intervention (with rationale) **. Not applicable**

10a Clinician and patient-assessed outcomes (if available). **Not applicable**

10b Important follow-up diagnostic and other test results **. Not applicable**

10c Intervention adherence and tolerability (How was this assessed?). **Not applicable**

10d Adverse and unanticipated events**. Not applicable**

Discussion

11a A scientific discussion of the strengths AND limitations associated with this case report. The case report highlights a rare and critical complication of severe malaria giant duodenal perforation in a pediatric patient, contributing valuable clinical insight to an underreported phenomenon.

Detailed clinical presentation, diagnostic workup, surgical intervention, and postoperative management are thoroughly described, providing a comprehensive view of the case.

The report includes exclusion of common peptic ulcer risk factors such as Helicobacter pylori infection, strengthening the argument for malaria as the causative factor.

Integration of relevant literature and comparison with similar rare cases from different geographic regions enhances the contextual understanding and generalizability.

Being a single case report, the findings have limited generalizability and cannot establish causality between malaria and giant duodenal perforation.

Lack of detailed histopathological examination of the duodenal tissue limits understanding of the exact pathological mechanisms involved.

Absence of long-term follow-up data restricts assessment of the patient's recovery trajectory and potential late complications. **Line: 156-172.**

11b Discussion of the relevant medical literature **with references.** The occurrence of giant duodenal perforation in this 5-year-old patient with severe malaria highlights a rare but critical complication of malaria in pediatric populations. While peptic ulcer disease (PUD) is predominantly linked to Helicobacter pylori infection and certain demographic factors such as blood group O and male gender, this case deviates from these common associations, suggesting alternative pathogenic mechanisms (Sullivan, 2010). The absence of H. pylori infection and the patient's blood group B status, along with her young age and female gender, indicate that the ulceration and subsequent perforation likely resulted from secondary causes rather than primary PUD (Goldman et al., 2012, Sullivan, 2010).

Secondary PUD in children is often precipitated by systemic stressors, including severe infections, shock, or inflammatory states, which can disrupt mucosal integrity and impair healing (Stabell et al., 2013, Tanzer et al., 1994, Wilson and Darby, 1990, Yadav et al., 2009). The pathogenesis of duodenal perforation in malaria is multifactorial, likely involving a combination of direct parasitic injury to the duodenal mucosa, shock and dehydration due to gastrointestinal fluid loss, and physiological stress induced by severe illness (Hua et al., 2007, Stabell et al., 2013, Wilson and Darby, 1990). This

pathophysiological cascade may increase the risk of ulcer development and, in rare cases, perforation. Evidence about duodenal perforation caused by malaria is scarce. Our case report, however, is in line with a study from Metema, Gondar, Ethiopia that found a 4-year-old child had a giant duodenal perforation linked to a malaria complication (Getu et al., 2025), while a study from Nepal reported a 5-year-old boy who had a duodenal perforation as a result of severe malaria (Bhandari et al., 2016). A similar instance of duodenal perforation in a 4-year-old boy was reported from India (Dewanda and Midya, 2015). Ghana's Kintampo Municipal Hospital reported a case involving a 1-year-old infant, highlighting the possibility of this problem even in younger patients (Goldman et al., 2012).

This case report presents a rare and significant occurrence of giant duodenal perforation secondary to severe malaria in a pediatric patient, highlighting an uncommon but critical gastrointestinal complication associated with malaria. While malaria predominantly manifests with febrile illness and hematologic complications, its association with peptic ulcer disease (PUD) and subsequent perforation remains scarcely reported, especially in children(Hua et al., 2007). The absence of typical risk factors for PUD in this patient such as Helicobacter pylori infection, blood group O, medication use, or underlying comorbidities strengthens the likelihood that the duodenal perforation was a direct complication of severe malaria.

The clinical presentation of peritonitis with abdominal distension, guarding, and rigidity in the context of severe malaria should prompt clinicians to consider gastrointestinal perforation as a differential diagnosis, particularly in endemic regions where malaria is prevalent.

Surgical intervention remains the cornerstone of management for duodenal perforation. In this case, exploratory laparotomy with primary repair using an omental pedicle patch and thorough peritoneal lavage was successfully performed followed by broad spectrum antibiotics plus targeted antimalarial therapy. Early surgical management combined with appropriate treatment of the underlying malaria infection is crucial to reduce morbidity and mortality associated with this complication.

This report underscores the need for heightened clinical vigilance for abdominal emergencies in children with severe malaria, as delays in diagnosis and treatment of perforation can lead to poor outcomes. It also broadens the clinical spectrum of malaria-related complications, advocating for the inclusion of secondary PUD and gastrointestinal perforation in the differential diagnosis of acute abdomen in pediatric patients from malaria-endemic areas.

Future studies are reasonable to better understand the incidence, pathogenesis, and optimal management strategies for malaria-associated gastrointestinal complications. Meanwhile, awareness among healthcare providers can facilitate timely diagnosis and intervention, improving prognosis in affected children.**Line:107-155.**

11c The scientific rationale for any conclusions (including assessment of possible causes) .This case report highlights a rare but serious complication of severe malaria in a pediatric patient, indicating that giant duodenal perforation can occur even in the absence of common risk factors for peptic ulcer disease. Prompt recognition of acute abdominal signs in children with severe malaria is essential to facilitate timely surgical intervention and appropriate antimalarial treatment, thereby reducing morbidity and mortality. Increased clinical awareness of gastrointestinal complications in malaria-endemic regions is crucial for improving outcomes in affected children.Line: **174-181.**

11d The primary "take-away" lessons of this case report (without references) in a one paragraph conclusion. This case report highlights a rare but serious complication of severe malaria in a pediatric patient, indicating that giant duodenal perforation can occur even in the absence of common risk factors for peptic ulcer disease. Prompt recognition of acute abdominal signs in children with severe malaria is essential to facilitate timely surgical intervention and appropriate antimalarial treatment, thereby reducing morbidity and mortality. **Line: 175-179.**

Patient Perspective **12** The patient should share their perspective in one to two paragraphs on the treatment(s) they receive . **Not applicable**

Informed Consent **13** Did the patient give informed consent? Please provide if requested . Written informed consent was obtained and signed by the patients' parents for publication and the use of any accompanying images. A copy of the written consent is available from the corresponding author upon request. **Line: 207-209**

Yes ☒ **No** ☐
